# Underlying Mechanisms of Increased Precipitation and Arbuscular Mycorrhizal (AM) Fungi on Plant Community by Mediating Soil Microbes in Desert Ecosystems

**DOI:** 10.3390/plants14213386

**Published:** 2025-11-05

**Authors:** Wan Duan, Hui Wang, Zhanquan Ji, Qianqian Dong, Wenshuo Li, Wenli Cao, Fangwei Zhang, Yangyang Jia

**Affiliations:** 1 College of Ecology and Environment, Xinjiang University, Urumqi 830046, China; duanwan1@stu.xju.edu.cn (W.D.); wanghui@stu.xju.edu.cn (H.W.); jizhanquan@stu.xju.edu.cn (Z.J.); dongqianqian@stu.xju.edu.cn (Q.D.); 19606365367@163.com (W.L.); caowenli@stu.xju.edu.cn (W.C.); 15033587081@163.com (F.Z.); 2 Key Laboratory of Oasis Ecology, Xinjiang University, Urumqi 830046, China

**Keywords:** increased precipitation, arbuscular mycorrhizal fungi, Gurbantunggut desert, phospholipid fatty acid

## Abstract

The increasing frequency of global extreme climate events has heightened the need to understand the mechanisms through which desert ecosystems respond to altered precipitation patterns. This includes elucidating how arbuscular mycorrhizal fungi (AMF) drive these responses by regulating key soil processes and shaping microbial community dynamics. We therefore conducted an in situ experiment involving increased precipitation and AMF suppression, and phospholipid fatty acid (PLFA) was employed to reveal the changes in soil microbial community. Results showed that increased precipitation significantly promoted the growth of soil AMF and Actinobacteria (Act). Increased precipitation significantly changed soil microbial community structure and promoted soil microbial community diversity, but it posed neutral effects on soil microbial community biomass. AMF suppression clearly inhibited AM fungal growth but increased the growth of Act and Gram-positive bacteria (G^+^) and posed limited effects on Gram-negative bacteria (G^−^), leading to an increased G^+^/G^−^ ratio. Notably, AMF suppression posed slight effects on the biomass, diversity, and structure of soil microbial community. Random forest analysis revealed that soil ammonium nitrogen (NH_4_^+^-N), microbial biomass nitrogen (MBN), and soil organic carbon (SOC) were the main factors influencing different soil microbes, and soil Act and G^+^ were the main factors influencing plant community diversity, but AMF were the primary factor influencing plant community biomass. More importantly, structural equation modeling (SEM) results further confirmed that increased precipitation and AMF significantly altered plant community diversity by influencing soil AM fungi and increased plant community biomass by promoting soil AM fungal growth. In conclusion, our results demonstrate that increased precipitation enhances plant community productivity and diversity in desert ecosystems primarily by stimulating the growth of arbuscular mycorrhizal fungi, which function as a key biological pathway mediating the ecosystem’s response to climate change.

## 1. Introduction

In many parts of the world, precipitation has undergone significant changes due to ongoing global warming [1,2]. Numerous datasets have indicated that annual precipitation showed a marked increasing trend with increased frequency and intensity of extreme precipitation events [3]. Both plant community structure and ecological functions of desert ecosystems exhibit high sensitivity to increased precipitation due to their low productivity and sparse vegetation cover, and they are extremely difficult to recover after environmental disturbances [4,5]. Soil water availability is recognized as the most critical limiting factor for plant growth and ecological functions in desert ecosystems [6,7]. Increased precipitation not only directly alleviates water stress but also selectively stimulates key moisture-sensitive functional groups, thereby driving the reorganization of soil microbial community structure [8,9]. Furthermore, soil functional microbes form strong symbiotic relationships with plants, play pivotal roles in plant–soil interactions by influencing nutrient cycling and uptake, and are key to maintaining plant community productivity and stability in desert ecosystems [10,11]. Therefore, the influence mechanisms of increased precipitation on desert plant communities through alterations in soil microbial communities have become a core research topic due to drastic changes in precipitation and require in-depth investigation.

Increased precipitation causes notable changes in soil microbial community structure through multiple pathways with distinct manifestations in arid environments [12,13]. First, in water-limited desert ecosystems, increased precipitation significantly promotes the decomposition of scarce soil organic matter and enhances nitrification in these inherently nutrient-poor soils [9]. For example, the enhanced nutrient availability, particularly nitrogen, leads to a significant increase in the relative abundance of r-strategist microbes (e.g., Gram-negative bacteria (G^−^)), which are highly efficient at utilizing transient resources. Conversely, K-strategist microbes (e.g., Gram-positive bacteria (G^+^)) tend to lose their competitive advantages due to their high tolerance to drought and low responses to environmental perturbations [14,15]. Previous studies in arid lands further indicate that, among these K-strategists, Actinobacteria (Act), in particular, experience a notable decline in their relative abundance in response to increased precipitation [16,17]. Second, in desert ecosystems, pH is a key environmental driver of microbial composition across arid and semi-arid regions, and precipitation-induced shifts in soil pH critically regulate the soil microbial community structure [18,19,20]. It is widely known that soil bacterial community diversity is positively correlated with soil pH ranging from 4 to 8, while soil fungal community diversity shows a negative correlation [21]. And soil bacterial communities tend to homogenize with the progression of soil acidification, while soil fungal communities exhibit increased heterogeneity [22,23]. This asymmetric response highlights the differential sensitivity of microbial functional groups to pH shifts in water-limited desert environments. Furthermore, in desert ecosystems, the most direct effect of increased precipitation on soil microbial communities is the alleviation of water stress, which thereby enhances soil microbial metabolic activity [24,25]. The increased activity of soil functional microorganisms, such as arbuscular mycorrhizal fungi (AMF), could greatly improve plant nutrient uptake efficiency, increase plant growth, and ecosystem stability [26,27]. Although soil microbes play important roles in nutrient cycling and plant growth, how soil functional microbes, combined with increased precipitation, affect soil microbial community structure remains a scientific issue that needs further clarification at present.

AMF belonging to the phylum Glomeromycota form mutualistic symbioses with the vast majority of terrestrial plants [28]. In desert ecosystems, the extensive hyphal networks of these symbionts significantly enhance their host plant’s capacity for water and nutrient acquisition (e.g., phosphorus and nitrogen), thereby promoting plant growth and increasing plant community diversity [29,30]. Furthermore, AMF create distinct microenvironments, selectively inhibit or promote specific soil microbial taxa, and subsequently alter soil microbial community structure [31,32]. For example, AMF hyphal residues provide complex organic polymers (e.g., chitin and cellulose), which serve as carbon sources for Gram-positive bacteria and thereby enhance their metabolic and competitive activities [33]. However, previous studies also demonstrated that AMF engage in strong resource competition with Gram-negative bacteria for soluble organic carbon, resulting in a decline in the abundance of Gram-negative bacteria [34]. In contrast, AMF exhibit mutualistic interactions with phosphate-solubilizing bacteria, such as *Pseudomonas*, in which AMF supply carbon to *Pseudomonas* via hyphal exudates, promote the relative abundance and activities of *Pseudomonas*, and subsequently increase soil phosphorus availability [35,36]. While the roles of AMF in maintaining plant community structure and ecological functions have been extensively studied, their roles in mediating plant and soil microbial communities in response to increased precipitation remain poorly understood, particularly in desert ecosystems. Thus, elucidating the influence mechanisms of AMF on soil microbial communities and cascading effects on plant communities is essential for building a robust scientific foundation to predict ecosystem responses and adaptive strategies in the face of increased precipitation.

Here, we conducted an in situ experiment by suppressing AMF activities under increased precipitation to investigate the roles of AMF in mediating soil microbial communities and cascading effects on plant communities in a desert ecosystem. Phospholipid fatty acid (PLFA), a biomarker technique that targets the membrane phospholipids of living microorganisms, was employed to characterize the soil microbial community structure and biomass. PLFA method provides a reliable assessment of the “live” soil microbial community, avoiding interference from DNA of dead soil microbes [37]. Considering this, PLFA method enables sensitive detection of changes in soil microbial community biomass and composition induced by environmental perturbations, including soil fungi, bacteria, and various functional groups [38,39]. Furthermore, desert ecosystems typically have low plant community biomass, sparse vegetation, and large areas of bare soil [40]. Considering this, desert ecosystems provide an important study area to uncover the real responses of soil microbial communities to increased precipitation without the interference of plant communities. This study was conducted in the largest mid-latitude arid region to address the following question: How do AMF affect plant communities through the regulation of soil microbial communities under increased precipitation in desert ecosystems?

## 2. Results

### 2.1. Responses of Soil Physicochemical Properties and Plant Community to Increased Precipitation and the Suppression of AMF

**Figure 1:**
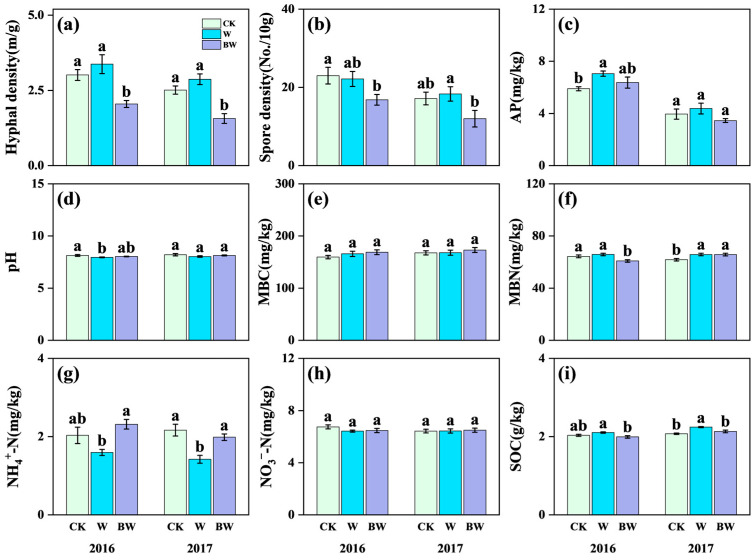
Effects of increased precipitation (W) and arbuscular mycorrhizal fungi suppression (BW) on soil physicochemical properties in 2016 and 2017. (**a**) Hyphal density; (**b**) spore density; (**c**) available phosphorus (AP); (**d**) pH; (**e**) microbial biomass carbon (MBC); (**f**) microbial biomass nitrogen (MBN); (**g**) ammonium nitrogen (NH_4_^+^-N); (**h**) nitrate nitrogen (NO_3_^−^-N); (**i**) soil organic carbon (SOC). CK, ambient precipitation treatment; error bars indicate ± SE. Different letters above the error bar indicate significant differences among treatments based on LSD’s post hoc test when *p* < 0.05. ANOVA results see Table S1.

First, AMF suppression significantly reduced spore density and hyphal density in 2016 and 2017; particularly, in 2017, AMF suppression treatment significantly reduced spore density by 46% and hyphal density by 35% (Figure 1a,b). These results indicated that the application of benomyl significantly suppressed soil AM fungal activities, which demonstrated that this method is effective for investigating the role of indigenous AM fungi in altering the soil microbial community. For soil physicochemical properties, increased precipitation and AMF suppression posed significant effects (Figure 1). Increased precipitation significantly reduced soil NH_4_^+^-N but increased SOC and MBN, and the effects were more pronounced in 2017 (Figure 1f,g,i; Table S1). In 2017, increased precipitation highly reduced soil NH_4_^+^-N by 34%, while increasing SOC and MBN by 8% and 7%, respectively (Figure 1f,g,i). Under increased precipitation, AMF suppression significantly increased soil NH_4_^+^-N but reduced SOC and MBN. Specifically, AMF suppression increased soil NH_4_^+^-N concentration by 45% in 2016 and by 40% in 2017 (Figure 1g). Conversely, AMF suppression significantly reduced SOC by 5% in both years (Figure 1i).

**Figure 2:**
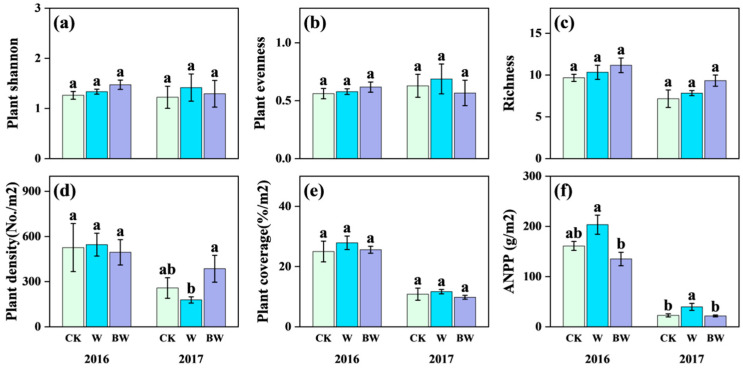
Effects of increased precipitation (W) and arbuscular mycorrhizal fungi suppression (BW) on plant community in 2016 and 2017. (**a**) Plant Shannon–Wiener diversity index; (**b**) plant Pielou’s evenness index; (**c**) richness; (**d**) plant density; (**e**) plant coverage; and (**f**) above-ground net primary productivity (ANPP). CK, ambient precipitation treatment; error bars indicate ± SE. Different letters above the error bar indicate significant differences among treatments based on LSD’s post hoc test when *p* < 0.05. ANOVA results see Table S2.

For the plant community, increased precipitation and AMF suppression had limited effects on species richness, plant Shannon diversity, plant evenness, and coverage in both years (Figure 2a–c,e; Table S3). Regarding plant community productivity, increased precipitation stimulated ANPP in both years, reaching statistical significance (*p* < 0.05) in 2017 with an increase of 74%. In contrast, under increased precipitation, AMF suppression significantly reduced ANPP but increased plant community density (Figure 2d,f), particularly in 2017. In 2017, AMF suppression significantly reduced ANPP by 45% (Figure 2f) and highly increased plant community density by 116% (Figure 2d).

### 2.2. Responses of Soil Microbial Community to Increased Precipitation and the Suppression of AMF

**Figure 3:**
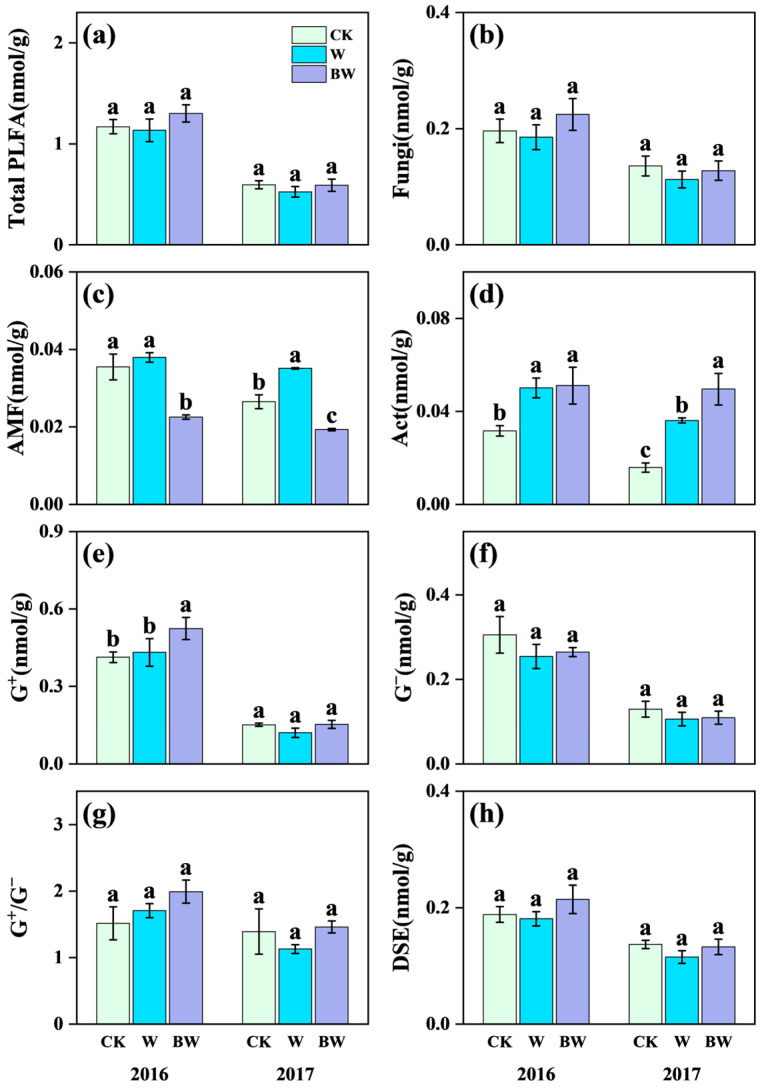
Effects of increased precipitation (W) and arbuscular mycorrhizal fungi suppression (BW) on microbial community in 2016 and 2017. (**a**) Total PLFA content of soil microbial community (Total PLFA); (**b**) fungi; (**c**) arbuscular mycorrhizal fungi (AMF); (**d**) Actinomycetes (Act); (**e**) Gram-positive bacteria (G^+^); (**f**) Gram-negative bacteria (G^−^); (**g**) the ratio of G^+^ (PLFA) and G^−^(PLFA)(G^+^/G^−^); (**h**) dark septate endophytes (DSE). CK, ambient precipitation treatment; error bars indicate ± SE. Different letters above the error bar indicate significant differences among treatments based on LSD’s post hoc test when *p* < 0.05. ANOVA results see Table S3.

Increased precipitation significantly promoted the growth of AMF and Act but had limited effects on soil fungi, G^+^, G^−^, dark septate endophytes (DSE), and total soil microbial biomass (Figure 3; Table S3). Specifically, increased precipitation increased AMF content by 33% in 2017 (Figure 3f). And increased precipitation highly increased the content of Act by 59% and 128% in 2016 and 2017, respectively (Figure 3d). Under increased precipitation, AMF suppression highly reduced the content of soil AMF; the magnitude of suppression reached 28% and 45% in 2016 and 2017, respectively (Figure 3f). These results further confirmed that benomyl application had notably inhibited effects on soil AMF. And AMF suppression promoted the growth of soil Act, with a significant increase of 37% in 2017 (Figure 3d). However, AMF suppression showed limited effects on the content of soil fungi, G^+^, G^−^, DSE, and total soil microbial biomass (Figure 3b,e,f,h; Table S3).

**Figure 4:**
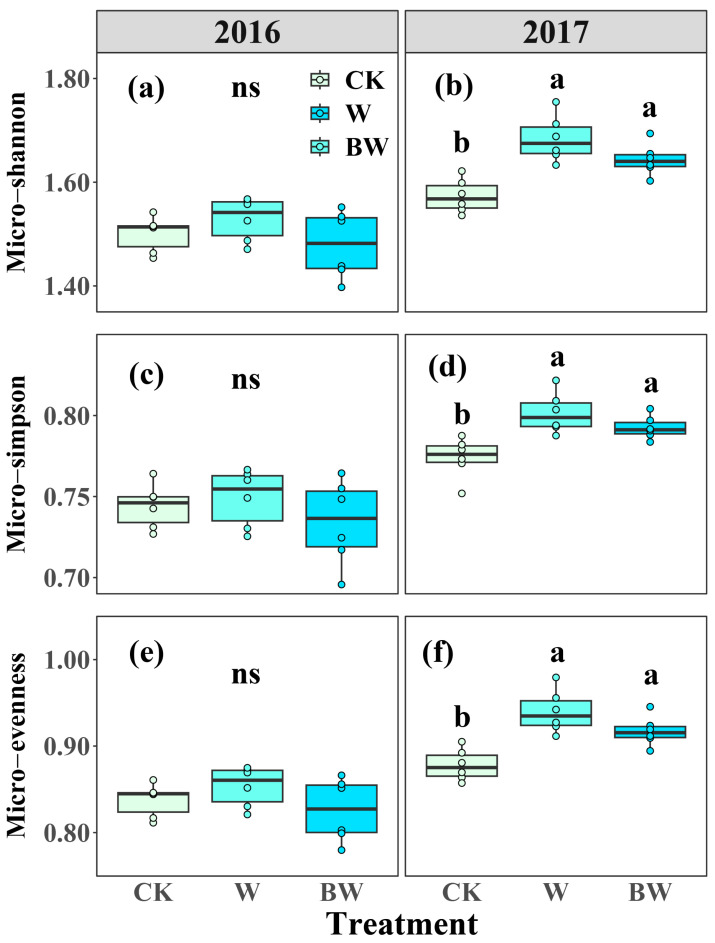
Effects of increased precipitation (W) and arbuscular mycorrhizal fungi suppression (BW) on microbial community diversity in 2016 and 2017. (**a**,**b**): Microbial Shannon–Wiener diversity index (Micro-Shannon); (**c**,**d**): microbial Simpson index (Micro-Simpson); (**e**,**f**): microbial Pielou’s evenness index (Micro-evenness). CK, ambient precipitation treatment; error bars indicate ± SE. Different letters above the error bar indicate significant differences among treatments based on LSD’s post hoc test when *p* < 0.05. ANOVA results see Table S3.

Analysis of the soil microbial community composition indicated that the combined relative abundance of DSE, G^+^, G^−^, and fungi collectively accounted for over 80% of the total soil community (Figure S1). Increased precipitation and AMF suppression led to a marginal decrease in the relative abundance of G^−^ in both years (Figure S1). In contrast, increased precipitation and AMF suppression increased the relative abundance of G^+^ in 2016 but had limited effects in 2017 (Figure S1). Increased precipitation promoted a slight increase in the relative abundance of Act and AMF in both years. However, AMF suppression resulted in a modest decrease in the relative abundance of Act and AMF in 2016 but led to a decrease in AMF and an increase in Act (Figure S1). For soil microbial community diversity and structure, increased precipitation increased micro-evenness, micro-Simpson, and micro-Shannon, with the effects reaching statistical significance in 2017 (Figure 4). Specifically, increased precipitation increased micro-Shannon, micro-Simpson, and micro-evenness by 7%, 4%, and 7%, respectively, in 2017 (Figure 4b,d,f). AMF suppression consistently tended to reduce soil microbial community diversity across both years but was not significant (Figure 4; Table S3). Based on the NMDS analysis, increased precipitation showed no effect on soil microbial community structure in 2016 but changed soil microbial community structure in 2017 (Figure S2). AMF suppression had limited effects on soil microbial community structure during the experimental period (Figure S3).

### 2.3. Underlying Mechanisms of Soil Microbial-Community-Mediated Effects of Increased Precipitation and AMF on Plant Community

**Figure 5:**
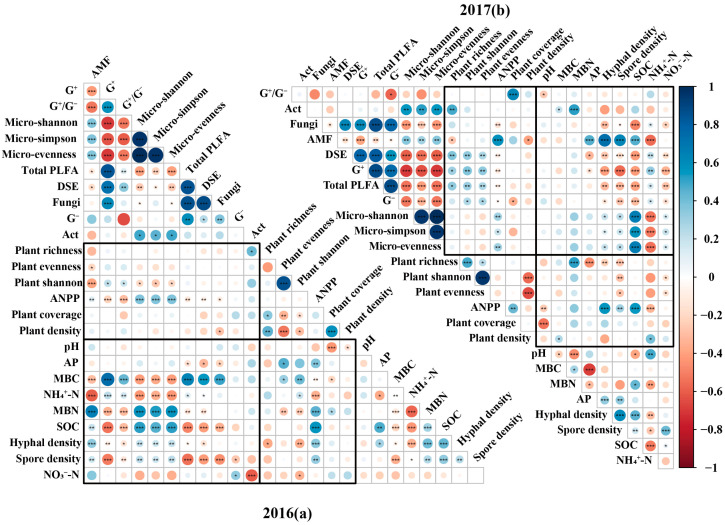
Correlation analysis among soil physicochemical properties, microbial communities, and plant communities in 2016 (**a**) and 2017 (**b**). Note: G^−^: Gram-negative bacteria; G^+^: Gram-positive bacteria; Act: Actinobacteria; AMF: arbuscular mycorrhizal fungi; G^+^/G^−^: the ratio of G^+^ (PLFA) and G^−^ (PLFA); Total PLFA: total PLFA content of soil microbial community; DSE: dark septate endophytes; Micro-Shannon: microbial Shannon–Wiener diversity index; Micro-Simpson: microbial Simpson index; Micro-evenness: microbial Pielou’s evenness index; AP: available phosphorus; MBC: microbial biomass carbon; MBN: microbial biomass nitrogen; NH_4_^+^-N: ammonium nitrogen; NO_3_^−^-N: nitrate nitrogen; SOC: soil organic carbon; Plant shannon: plant Shannon–Wiener diversity index; Plant evenness: plant Pielou’s evenness index; ANPP: above-ground net primary productivity. *, *p* < 0.05; **, *p* < 0.01; ***, *p* < 0.001.

Based on RDA analysis, SOC consistently emerged as one of the most important factors influencing soil microbial community structure. Its effect was more pronounced in 2017 (Figure S4). Specifically, correlation analysis revealed that SOC exhibited a highly significant negative correlation with AMF, while it showed highly significant positive correlations with Gram-positive bacteria, DSE, and fungi (Figure 5). In contrast, SOC demonstrated no substantial relationship with Gram-negative bacteria (Figure 5). RDA analysis further indicated that soil NO_3_^−^-N was identified as one of the primary factors shaping the soil microbial community in 2016 (Figure S4a); soil NH_4_^+^-N became a significant influencing factor for the soil microbial community in 2017 (Figure S4a). Based on correlation analysis, soil NO_3_^−^-N showed a significant positive correlation with Act and a significant negative correlation with G^−^ and showed neutral correlations with other functional microbial groups (Figure 5). Soil NH_4_^+^-N correlated positively and significantly with AMF but exhibited significant negative correlations with most other functional microbial groups, except for Act (Figure 5).

For plant Shannon and ANPP, RDA results revealed that AMF consistently emerged as one of the most significant indicators influencing the plant community (Figure S2). Correlation analysis confirmed that AMF exhibited a significant positive correlation with ANPP in both years (Figure 5). However, the relationship between AMF and plant diversity indices showed interannual variation. AMF was significantly and negatively correlated with plant Shannon and evenness in 2016. In contrast, a significant negative correlation was observed only with plant richness, while its relationships with plant Shannon and evenness were not significant in 2017 (Figure 5). Correlation analysis further indicated that G^+^ showed a significant positive correlation with plant Shannon but a negative correlation with ANPP (Figure 5). G^−^ showed no significant correlations with any of the measured plant community indicators in 2016 (Figure 5a) but were significantly and negatively correlated with ANPP and positively correlated with plant Shannon in 2017 (Figure 5b). Correlation analysis further indicated that Act maintained a significant positive correlation with plant richness but showed no significant relationships with other plant community metrics (Figure 5).

**Figure 6:**
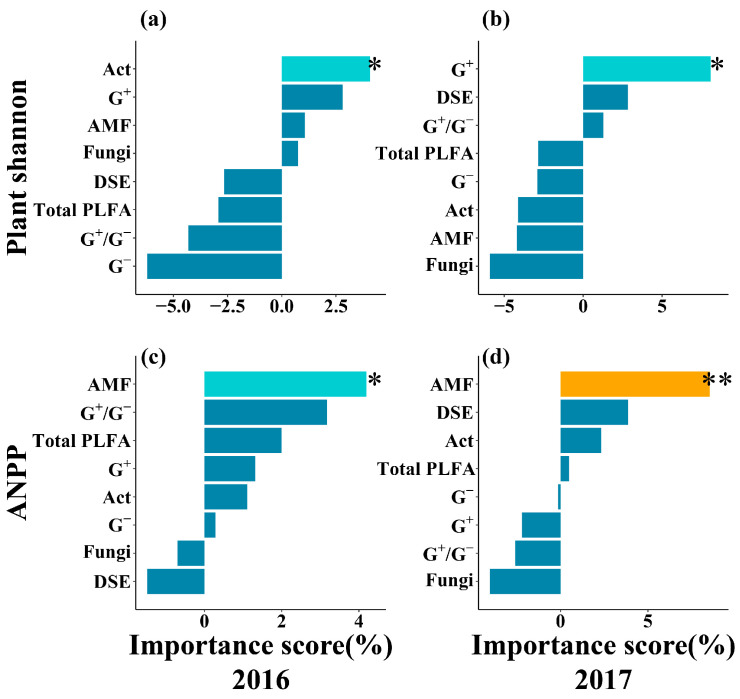
Relative importance of microbial community for predicting the plant community from random forest analysis. (**a**,**b**): Plant Shannon–Wiener diversity index (Plant Shannon); (**c**,**d**): above-ground net primary productivity (ANPP). G^−^: Gram-negative bacteria; G^+^: Gram-positive bacteria; Act: Actinobacteria; AMF: arbuscular mycorrhizal fungi; G^+^/G^−^: the ratio of G^+^ (PLFA) and G^−^(PLFA); Total PLFA: total PLFA content of microbial communities; DSE: dark septate endophytes. *, *p* < 0.05; **, *p* < 0.01.

Random forest analysis revealed that AMF were a significant predictor of ANPP in both years (Figure 6c,d). Concurrently, G^+^ emerged as one of the most important predictors for the plant Shannon–Wiener diversity index, reaching statistical significance in 2017 (Figure 6a,b). Furthermore, the results indicated that NH_4_^+^-N and SOC consistently ranked among the most important predictors for AMF and G^+^, respectively, with their influence also becoming statistically significant in 2017 (Figure S5).

**Figure 7:**
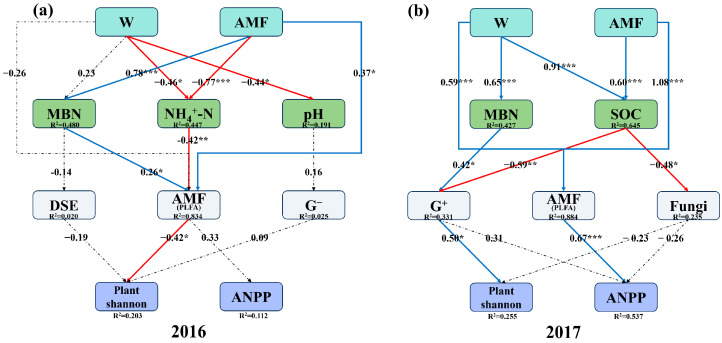
Structural equation modeling (SEM) showing the causal pathways by which arbuscular mycorrhizal fungi (AMF) and increased precipitation (W) influence plant community in 2016 and 2017. (**a**) *p* = 0.381; RMSEA = 0.057; GFI = 0.847; AIC = 15.266; df = 28. (**b**) *p* = 0.174 RMSEA = 0.125; GFI = 0.976; AIC = 0.638; df = 21. MBN: microbial biomass nitrogen; NH_4_^+^-N: ammonium nitrogen; SOC: soil organic carbon; DSE: dark septate endophytes; G^−^: Gram-negative bacteria; G^+^: Gram-positive bacteria; Plant Shannon: plant Shannon–Wiener diversity index; ANPP: above-ground net primary productivity. Red and blue solid arrows indicate negative and positive significant effects, respectively, and black dashed lines indicate non-significant effects. The numbers above the arrows indicate the magnitude of the standardized SEM coefficients (*, *p* < 0.05; **, *p* < 0.01; ***, *p* < 0.001). R^2^ values represent the proportion of variance explained for each variable.

SEM results revealed that, in 2016, increased precipitation significantly promoted AMF via NH_4_^+^-N, which subsequently led to a significant suppression of plant Shannon while promoting ANPP (Figure 7a). In 2017, the pathway shifted; increased precipitation directly exerted a significant promotional effect on AMF, which subsequently significantly enhanced ANPP (Figure 7b). Also in 2017, increased precipitation exerted contrasting effects on G^+^ through two distinct pathways, a positive effect via MBN and a negative effect via SOC, with the SOC pathway demonstrating a stronger influence (Figure 7b). Specifically, both increased precipitation and AMF collectively inhibited G^+^ primarily through their positive effect on SOC. This suppression of G^+^ was consequential, as G^+^ itself significantly promoted plant Shannon (Figure 7b).

## 3. Discussion

### 3.1. The Influence Mechanisms of Increased Precipitation on Soil Microbial Community

Increased precipitation significantly altered the structure and composition of soil microbial communities, which has been demonstrated in diverse ecosystems [41,42]. Our results further revealed that the responses of different soil functional groups differed, and the effects of increased precipitation on soil microbial communities differed under different background precipitation levels. Specifically, increased precipitation promoted the growth of AMF, with a more pronounced effect in 2017, which was a natural precipitation deficiency, and the underlying mechanisms differed between the two years (Figure 3c). Increased precipitation promoted soil AMF by exerting a negative effect on soil NH_4_^+^-N, which was consistent with previous studies (Figure 7a). This is because elevated soil moisture intensified nitrification, promoting the conversion of NH_4_^+^-N to NO_3_^−^-N, thereby alleviating the negative impact of high NH_4_^+^-N on AMF colonization [43,44]. In contrast, in water-stressed year conditions, increased precipitation directly alleviated water limitation, thereby promoting AMF growth (Figure 3c and Figure 7b) [45,46]. Furthermore, the changes in soil moisture induced by increased precipitation also significantly impacted soil bacterial community structure [14,15]. In the present study, increased precipitation inhibited Gram-positive bacteria through the promoting effects on SOC and decreased the G^+^/G^−^ ratio (Figure 3g and Figure 7b), which was contrary to one most recent study [47]. The possible reason is that desert ecosystems are more sensitive to carbon input, and soil microbes experience more intense competition compared to forest ecosystems [48]. Increased precipitation could potentially enhance plant-derived carbon input, leading to a rise in the proportion of soil labile organic carbon [49]. Gram-positive bacteria prefer to utilize recalcitrant soil organic carbon [16], whereas Gram-negative bacteria are more efficient in decomposing labile organic carbon, resulting in Gram-positive bacteria being at a competitive disadvantage [17]. Notably, as a member of Gram-positive bacteria, Actinobacteria were significantly promoted by increased precipitation (Figure 3d), which was consistent with a previous study [50]. The possible reason is that Actinobacteria with a unique branched filamentous structure enable them to expand their living space and improve the ability of absorbing water and taking up nutrients, thus conferring them a stronger competitive advantage compared to other Gram-positive bacteria [51].

Furthermore, our results further demonstrated that increased precipitation increased soil microbial community diversity, significantly altered soil microbial community structure, but posed limited effects on soil microbial community biomass. These findings were consistent with previous studies, which found that increased precipitation significantly alters the microbial community structure but has no significant impact on the soil microbial biomass in alpine meadows [52,53]. As discussed above, different soil microbes responded differently to increased precipitation, which certainly would cause changes in soil microbial community structure. Moreover, increased precipitation could effectively activate dormant microbial spores, prompting more functional groups to become active and thereby increasing the diversity of the soil microbial community [45,54]. The limited effects on soil microbial biomass are likely due to persistent nutrient (C and N) limitations in these barren desert soils, which are not fully alleviated by a limited increase in precipitation [9,55,56]. In summary, increased precipitation posed significant effects on soil microbial communities, mainly through influencing soil NH_4_^+^-N, NO_3_^−^-N, and SOC. Considering the relatively short-term experimental period, long-term observations should be conducted to generalize our findings to other ecosystems.

### 3.2. The Influence Mechanisms of Arbuscular Mycorrhizal Fungi on Soil Microbial Community

The obvious effect of AMF suppression on the soil microbial community was reflected by a significant reduction in AMF-specific PLFA content. This suppression was further corroborated by the simultaneous decrease in soil hyphal density and spore density. These results provided solid evidence that benomyl application is still an effective and direct method for suppressing AM fungal activity in natural conditions, which has been widely used in previous studies [57,58,59]. Meanwhile several previous studies demonstrated that benomyl application has the risk of affecting the activities of other soil microbes, such as pathogenic fungi [60,61]. For instance, benomyl application might reduce the fecundity of ants and alter the composition of ant assemblages [60]. While existing studies revealed that benomyl application highly inhibited AM fungal growth and reduced PLFA content, hyphae density, and spore density, without disturbing the other soil fungal PLFA concentrations, which were consistent with our findings [62,63]. And the changes in the soil microbial community were due to the suppression of AM fungal activities.

Our study provided clear in situ evidence that AMF play an important role in altering the soil microbial community structure. AMF significantly inhibited Gram-positive bacteria growth but showed no significant effect on Gram-negative bacteria, leading to a decrease in the G^+^/G^−^ ratio (Figure 3g), a pattern consistent with a previous study [64]. This phenomenon may follow a mechanism which is similar to the effects of increased precipitation on Gram-positive bacteria. AMF inhibited Gram-positive bacteria growth through promoting soil organic carbon, as indicated by our SEM results. In contrast, Gram-negative bacteria can not only form mutualistic relationships with AMF to activate soil nitrogen and phosphorus nutrients but also complement the utilization of labile organic carbon by AMF [65]. This could be the reason why they were not inhibited. Notably, unlike the findings reported by Wang et al. (2023) [66], AMF in this study did not exhibit a promoting effect on Actinobacteria. Instead, inhibitory effects on Actinobacteria were observed. One possible reason is that nutrient stress forced AMF to prioritize survival, shifting the relationship with Actinobacteria from potential mutualism to direct competition for limited nutrients, particularly in desert ecosystems [67].

Additionally, although we found significant effects of AMF on soil microbial community structure, limited effects of AMF on soil microbial community biomass and diversity were observed. In this study, AMF suppression tended to increase soil microbial community biomass (Figure 3a), which was consistent with the findings of Welc et al. [68]. One possible reason is that AMF, through its symbiotic relationship with plants, maintains a competitive advantage over other soil microbes, particularly in water- and nutrient-limited ecosystems [69]. By utilizing an extensive mycorrhizal network to absorb soil nutrients more efficiently and broadly, AMF may control the activities of other soil microbes and increase overall soil microbial biomass after AMF suppression [68]. Meanwhile, despite the inhibitory effects of AMF on the growth of most soil functional microbes, AMF tended to increase soil microbial community diversity. This may be because the mycorrhizal network created a mosaic of diverse microhabitats, providing more niches and living space for soil microbial species with different resource utilization strategies [70]. This allows a more diverse array of soil microbial taxa to coexist, thereby enhancing soil microbial community diversity.

### 3.3. The Influence Mechanisms of Increased Precipitation and Arbuscular Mycorrhizal Fungi on Plant Community Through Altering Soil AMF and G^+^

The most interesting finding of our study is perhaps that increased precipitation and AMF suppression influence plant communities by altering soil microbes, particularly AMF and G^+^. Increased precipitation was demonstrated to promote plant community biomass through direct or indirect regulatory effects on AMF, with AMF being identified as the most critical predictor of ANPP. It is widely known that AMF can form tight relationships with desert plants, increase plant growth by promoting nutrient uptake, and maintain stable plant community biomass under environmental perturbations [9,10]. As demonstrated by previous studies, even in years with varying precipitation conditions, plants could still efficiently utilize the mycorrhizal network to enhance the efficiency of absorbing soil water and nutrients, with AMF consistently exhibiting excellent and stable ecological functions [29,30,71]. Furthermore, our results revealed that increased precipitation enhanced plant community diversity by alleviating water stress and increasing nutrient availability, whereas AMF reduced species richness, with the effects depending on the background of natural precipitation levels (Figure 2a,c). In normal precipitation (i.e., 2016), AMF exhibited a significant negative effect on plant community diversity (Figure 2a). This may be because mycorrhizal plants with a mutualistic relationship with AMF demonstrated high efficiency in utilizing soil water and nutrients through the mycorrhizal network, outcompeting non-mycorrhizal plants and subsequently reducing plant community diversity [72]. In contrast, during the relatively dry year (i.e., 2017), AMF increased community evenness and Shannon–Wiener diversity (Figure 2a,b). The reason could be that AMF enhanced plant water and nutrient uptake, supported the survival of non-dominant species with high fungal dependency, and significantly increased community evenness despite a decrease in species richness [73].

Additionally, our results further indicated that increased precipitation and AMF affected plant community diversity by promoting SOC, which inhibited Gram-positive bacteria (Figure 7b). Gram-positive bacteria were among the most important factors influencing plant Shannon diversity (Figure 6a,b), with SOC being one of the most critical predictors for Gram-positive bacterial abundance (Figure S5k,l). These findings are consistent with previous studies [74]. One possible reason could be that Gram-positive bacteria are primary producers of antibiotics and secondary metabolites in soil. Their decreased abundance significantly reduces the soil’s disease-suppressive capacity, allowing pathogenic microorganisms to proliferate, leading to increased plant diseases and ultimately a decline in plant diversity [75]. In summary, our results highlight the important role of AMF in altering the plant community under environmental perturbations in desert ecosystems. And our results call for future research to consider various ecosystem types with multiple climate change factors to generalize the roles of AMF in altering plant communities.

## 4. Materials and Methods

### 4.1. Study Site

This study was carried out in the Gurbantunggut Desert, which is located in the central part of the Junggar Basin, northwest China (44°15′–46°50′ N, 84°50′–91°20′ E). This desert is recognized as the largest fixed and semi-fixed desert in China, which is predominantly composed of fixed and semi-fixed dunes [76]. The area has a typical temperate continental desert climate, characterized by hot summers and cold winters due to its enclosed topography and atmospheric circulation patterns [77]. The mean annual temperature and precipitation are approximately 7.19 °C and 215.6 mm, respectively [78]. The average annual snow cover period lasts between 100 and 150 days with more than 20 cm of snow [79]. The soils are gray desert soils (Chinese classification) with aeolian sands on the surface (0–100 cm), fine sand particles (0.05–0.25 mm) constitute the dominant fraction (>85%), while the content of silt and clay particles (<0.002 mm) is extremely low (<5%). Accordingly, the soils are extremely nutrient-poor with low organic matter content [80]. The vegetation consists of a mixture of shrubs and grasses. *Haloxylon ammodendron* and *Haloxylon persicum* are the constitutive species in the desert community; annual herbs, such as *Erodium oxyrrhynchum*, are distributed in the understory of the shrubs [81,82].

### 4.2. Experimental Design

A typical and representative study site was selected in the southern Gurbantunggut Desert and was isolated by steel fences to prevent herbivory before the plants germinated in 2014. A two-factor experiment was established, involving ambient precipitation (CK), simulating increased precipitation (W), and benomyl application with increased precipitation (BW). The experimental setup consisted of 18 plots (2.5 × 2.5 m), with a 2 m buffer zone between any two plots. Each treatment had six replicates. Each main plot was divided into four 1 × 1 m subplots. The increased precipitation treatment was based on projections indicating a potential increase of 40 mm in annual precipitation over the next 50 to 100 years [83,84]. Therefore, the increased precipitation treatment was applied to add a total of 40 mm of water to the natural precipitation. Water was added every two weeks during the growing season for a total of four applications. For each application, the subplots in the W treatment received 10 L of water (equivalent to 10 mm of rainfall). For benomyl application treatment, benomyl was used to effectively suppress AMF activity with negligible effects on non-target fungi [59,85]. Benomyl was applied as an aqueous solution at a concentration of 0.6 g of the active ingredient per liter of water [59,86]. In addition, benomyl must be applied with water to suppress AMF activity, so we do not have a benomyl treatment without water. The solution was uniformly sprayed using a powered sprayer to ensure optimal effects.

Plant samples were collected in late May of 2016 and 2017, when plant biomass reached its maximum. All plants within each subplot were identified and classified into their aboveground and root components. Concurrently, plant community metrics such as coverage, density, and species richness were surveyed, with the Simpson index and Shannon–Wiener diversity index calculated afterward. After returning to the laboratory, plant materials were oven-dried at 65 °C for 72 h to measure primary productivity. For soil samples, three sampling points were selected in each sampling area. After removing the litter layer, surface soil (0–20 cm) was collected using a 3 cm diameter soil corer. The samples from each subplot were sieved through a 2 mm sieve and then mixed thoroughly. In total, 18 composite samples were taken per year (3 treatments × 6 replicates), for a total of 36 samples in two years. The soil samples were then divided into four parts: one part was naturally dried for the determination of soil physicochemical properties, the second portion was immediately placed in sterilized polyethylene bags and stored in a refrigerator at 4 °C until the soil microbial carbon and N were determined within one month, the third part was stored in a refrigerator at −20 °C for soil nitrate nitrogen (NO_3_^−^-N) and ammonium nitrogen (NH_4_^+^-N) analysis, and the fourth was stored at −80 °C for PLFA analysis. Notably, during our sampling years (2016 and 2017), there was a significant difference in annual precipitation: 203.7 mm in 2016 compared to 126.8 mm in 2017. In contrast, the average annual temperatures were very similar at 6.3 °C in 2016 and 6.1 °C in 2017.

### 4.3. PLFA Determination and Plant Community and Soil Parameters Measurements

The diversity indices for both the plant and soil microbial communities were calculated, i.e., Shannon–Wiener diversity index (*H*) was calculated by the formula $H=-\sum_{i=1}^{N}P_{i}\ln P_{i}$ [87], Simpson index (*S*) was calculated by the formula $D\;=\;1-\sum_{i=1}^{N}{P_{i}}^{2}$ [88], and Pielou’s evenness (*J*) was calculated by the formula $J\;=\;(\frac{1}{S})/N$ [89]. For the plant community, in the formulas, *N* represents the species richness of each plot, and pi represents the proportion of the *i*-th species to the total. For the microbial community (detailed information follows), in the formulas, the proportion of the *i*-th characteristic PLFA is defined as its ratio to the total number of fatty acids, and *S* represents the total number of PLFA types in the community.

For soil parameters, soil pH was determined by extracting soil samples with deionized water at a soil-to-water ratio of 1:5, followed by measurement with a pH meter. Soil organic carbon (SOC) was measured using the potassium dichromate–sulfuric acid oxidation method [90]. NH_4_^+^-N and nitrate nitrogen (NO_3_^−^-N) were analyzed using a flow analyzer [91]. Available phosphorus (AP) was determined using the acid-soluble molybdenum-antimony anti-colorimetric method [92]. And soil microbial biomass carbon (MBC) and nitrogen (MBN) were measured using the chloroform fumigation-K_2_SO_4_ extraction method [93]. For spore density assessment, 2.5 g of soil was weighed and centrifuged to remove debris [94]. The osmotic pressure was then adjusted by adding a 60% (*w*/*v*) sucrose solution, followed by another centrifugation and sieving step. After rinsing off the sucrose solution, the remaining material on the sieve was transferred to a Petri dish and observed under a stereomicroscope for counting. For hyphal density measurement, 2 g of soil was weighed, homogenized, stirred, and sieved [95]. The sample was subsequently vacuum-filtered through a 0.45 μm microporous membrane three times. Trypan blue staining solution was then added for staining, followed by the addition of lactophenol glycerol as a mounting medium and preservative. Under a 200× microscope, 25 random fields were uniformly sampled using the grid-intersect method.

For PLFA analysis, 8 g freeze-dried soil was weighed and extracted twice with a 0.8:1:2 (*v*/*v*/*v*) mixture of citrate buffer, chloroform, and methanol by vigorous shaking. The extracted fatty acids were then separated using a Solid Phase Extraction (SPE) silica gel column. The recovered PLFAs were subsequently subjected to base-catalyzed methylation, with n-methyl nonadecanoate (19:0) as the internal standard, and analyzed using a Gas Chromatograph equipped with a Flame Ionization Detector (GC-FID; Agilent 7890B, Santa Clara, CA, USA). Quantitative analysis was performed using fatty acid standards and the microbial identification system (MIDI, Inc., Newark, DE, USA), with results expressed in nanomoles per gram of soil (nmol/g) [96,97]. According to PLFA analysis method [97,98], the results were classified into six major microbial groups based on specific PLFA biomarkers: Gram-positive bacteria (i12:0, i13:0, i14:0, a15:0, i15:0, i16:0, a16:0, a17:0, i17:0, i18:0), Gram-negative bacteria (i15:0 3OH, 16:1ω7c, 16:1ω9c, 17:1ω7c, 17:1ω8c, i17:0 3OH, 18:1ω7c, cy17:0), Actinobacteria (10Me16:0, 10Me17:0, 10Me18:0), fungi (18:1ω9c, 18:2ω6,9, 18:3ω6c(6,9,12)), arbuscular mycorrhizal fungi (16:1ω5c), and dark septate endophytes (DSE) (16:0, 18:0). For the soil microbial community diversity, i.e., Shannon–Wiener diversity, Simpson index, and Pielou’s evenness, the calculated formula was the same with plant community diversity, which was mentioned above.

### 4.4. Statistical Analysis

Initially, a one-way ANOVA was used to separately explore the effects of increased precipitation (increased precipitation treatment (W) compared to the control treatment (CK) and AMF suppression (benomyl application with increased precipitation (BW) compare to the increased precipitation treatment (W))) on soil physicochemical properties, content of each soil functional groups, and soil and plant community diversity indices. ANOVA was performed using SPSS 19.0, with the LSD test applied for pairwise comparisons at a significance level of *p* < 0.05 to identify specific significant differences among treatment groups. Subsequently, bar charts and stacked bar charts were used to illustrate the percentage changes in soil microbial community components, which were generated in the Origin Pro Student Version software. Non-metric Multidimensional Scaling (NMDS) analysis was employed to assess the effects of increased precipitation and AMF suppression on soil microbial structure, which was conducted in R 4.5.0 utilizing the “ggsignif” and “vegan” packages. Secondly, correlation analyses were created to explore the correlations among the plant community, soil microbial community, and soil physicochemical properties, which were conducted in the Origin Pro Student Version. And Redundancy Analysis (RDA) was performed to investigate the primary factors driving these relationships in the “vegan” package in R 4.5.0. Thirdly, a random forest model was established to quantify the contribution rates of individual soil physicochemical parameters to PLFA content of each soil functional group and the contribution rates of each soil functional group to plant Shannon diversity and above-ground net primary productivity (ANPP), using the “randomForest” package in R 4.5.0. Finally, a priori structural equation model (SEM) was performed to analyze hypothetical pathways that may explain how increased precipitation and AMF suppression influence plant community through altering soil physicochemical properties and soil functional groups. The SEM was constructed using the “lavaan” package in R 4.3.3 and conceptual diagrams visually represented in PowerPoint (Microsoft Office Home and Student 2019).

## 5. Conclusions

Increased precipitation caused considerable changes in the plant and soil microbial communities [53,99]. This study conducted a four-year in situ experiment with establishment of benomyl application with increased precipitation to simulate future precipitation scenarios in the Gurbantunggut Desert of Central Asia. PLFA technique was employed to demonstrate changes in soil microbial community structure and uncover the influence of patterns of increased precipitation and AMF on the soil microbial community. The results showed that increased precipitation significantly promoted the growth of soil AMF and Act, altering soil microbial community structure. And increased precipitation significantly enhanced soil microbial community diversity but had no effect on soil microbial community biomass. AMF suppression clearly inhibited AM fungal growth but increased the growth of Act and G^+^. Notably, AMF suppression posed limited effects on the biomass, diversity, and structure of the soil microbial community. Additionally, random forest analysis further indicated that soil NH_4_^+^-N, NO_3_^−^-N, MBC, MBN, and SOC were the main influencing factors on different soil microbes. Specifically, increased precipitation promoted the growth of AMF by inhibiting soil NH_4_^+^-N content, while significantly promoting the relative abundance of actinobacteria; however, increased precipitation posed negative effects on Gram-positive bacteria by promoting SOC. AMF inhibited soil fungi and G^+^ by increasing SOC. Furthermore, random forest analysis revealed that soil Act and G^+^ were the main influencing factors on plant community diversity, but AMF were the primary influencing factor on plant community biomass. Finally, SEM results further confirmed that increased precipitation and AMF significantly altered plant community diversity by influencing AM fungi, and increased precipitation and AMF increased plant community biomass by promoting soil AM fungal growth. Overall, this study revealed the complex mechanisms by which increased precipitation and AMF jointly regulated soil microbial community structure and subsequently affected plant community in desert ecosystems. These findings provide a new theoretical basis for understanding how desert ecosystems respond to global change and offer important practical references for ecological restoration and adaptive management in arid regions.

## Data Availability

Data are contained within this article and Supplementary Materials.

## Supplementary Materials

The following supporting information can be downloaded at: https://www.mdpi.com/article/10.3390/plants14213386/s1, Table S1: Results of two repeated-measures ANOVA on the effects of increased precipitation (W) and arbuscular mycorrhizal fungi suppression (BW) with the hyphal density, spore density, available phosphorus (AP), pH, microbial biomass carbon (MBC), microbial biomass nitrogen (MBN), ammonium nitrogen (NH_4_^+^-N), nitrate nitrogen (NO_3_^−^-N), and soil organic carbon (SOC) from 2016 to 2017; Table S2: Results of two repeated-measures ANOVA on the effects of increased precipitation (W) and arbuscular mycorrhizal fungi suppression (BW) with the plant Shannon–Wiener diversity index (Plant Shannon), Pielou’s evenness index (Plant evenness), richness, plant density, plant coverage, and above-ground net primary productivity (ANPP) from 2016 to 2017; Table S3: Results of two repeated-measures ANOVA on the effects of increased precipitation (W) and arbuscular mycorrhizal fungi suppression (BW) with the total PLFA content of soil microbial community (Total PLFA), Gram-negative bacteria (G^−^), Gram-positive bacteria (G^+^), actinomycetes (Act), fungi, arbuscular mycorrhizal fungi (AMF), G^+^/G^−^ (the ratio of G^+^ (PLFA), G^−^(PLFA)), dark septate endophytes (DSE), microbial Shannon–Wiener diversity index (Micro-Shannon), microbial Simpson index (Micro-Simpson), microbial Pielou’s evenness index (Micro-evenness) from 2016 to 2017; Figure S1: Effects of increased precipitation (W) and arbuscular mycorrhizal fungi suppression (BW) on relative abundance of microbial community components. CK: ambient precipitation treatment. G^−^: Gram-negative bacteria; G^+^: Gram-positive bacteria; Act: Actinobacteria; AMF: arbuscular mycorrhizal fungi; Figure S2: RDA analysis of microbial communities on plant communities in 2016 (a) and 2017 (b); Figure S3: NMDS analysis of increased precipitation (W) and arbuscular mycorrhizal fungi suppression (BW) on microbial community structure in 2016 (a) and 2017 (b); Figure S4: RDA analysis of soil physicochemical properties on microbial communities in 2016 (a) and 2017 (b); Figure S5: Relative importance of soil physicochemical properties for predicting the microbial community from random forest analysis.
