# Supplementary material for: Underlying Mechanisms of Increased Precipitation and Arbuscular Mycorrhizal (AM) Fungi on Plant Community by Mediating Soil Microbes in Desert Ecosystems
Source: Plants (Basel). 2025 Nov 5;14(21):3386. doi: 10.3390/plants14213386 (PMC12608211; doi:10.3390/plants14213386)

Table S1. Results of two repeated-measures ANOVA on the effects of increased precipitation (W) and arbuscular mycorrhizal fungi suppression (BW) with the hyphal density, spore density, available phosphorus (AP), pH, Microbial biomass carbon (MBC), Microbial biomass nitrogen (MBN), ammonium nitrogen (NH_4^+-N), nitrate nitrogen (NO_3^--N) and soil organic carbon (SOC) from 2016 to 2017.

Years	Treat-ment	Hyphal density (No./10g)	Spore density (m/g)	AP (mg/kg)	pH	MBC (mg/kg)	MBN (mg/kg)	NH_4^+N (mg/kg)	NO_3^- N (mg/kg)	SOC (g/kg)
2016	W	0.262	0.752	**$p < 0.05$**	**$p < 0.05$**	0.334	0.311	0.054	0.106	0.068
	BW	**$p < 0.01$**	0.057	0.111	0.34	0.625	**$p < 0.01$**	**$p < 0.01$**	0.792	**$p < 0.01$**
2017	W	0.13	0.666	0.385	0.161	0.971	**$p < 0.05$**	**$p < 0.001$**	0.964	**$p < 0.001$**
	BW	**$p < 0.001$**	**$p < 0.05$**	0.072	0.404	0.447	0.96	**$p < 0.01$**	0.794	**$p < 0.01$**

Table S2. Results of two repeated-measures ANOVA on the effects of increased precipitation (W) and arbuscular mycorrhizal fungi suppression (BW) with the Plant Shannon-Wiener diversity index (Plant shannon), Pielou's evenness index (Plant evenness), Richness, Plant density, Plant coverage, and above-ground net primary productivity (ANPP) from 2016 to 2017.

Years	Treatment	Plant shannon	Plant evenness	Richness	Plant density (No./m^2)	Plant coverage (%/m^2)	ANPP (g/m^2)
2016	W	0.506	0.753	0.535	0.906	0.417	0.055
	BW	0.212	0.489	0.439	0.757	0.518	**$p < 0.01$**
2017	W	0.599	0.718	0.533	0.408	0.656	**$p < 0.05$**
	BW	0.736	0.462	0.171	**$p < 0.05$**	0.324	**$p < 0.05$**

Table S3. Results of two repeated-measures ANOVA on the effects of increased precipitation (W) and arbuscular mycorrhizal fungi suppression (BW) with the total PLFA content of soil microbial community (Total PLFA), Gram-negative bacteria (G^-), Gram-positive bacteria (G^+), actinomycetes (Act), Fungi, arbuscular mycorrhizal fungi (AMF), G^+/G^- (the ratio of G^+ (PLFA), G^-(PLFA)), Dark Septate Endophytes (DSE), Microbial Shannon-Wiener diversity index (Micro-shannon), Microbial Simpson index (Micro-simpson), Microbial Pielou's evenness index (Micro-evenness) from 2016 to 2017.

Years	Treatment	Total PLFA (noml/g)	Fungi (noml/g)	AMF (noml/g)	Act (noml/g)	G^+ (noml/g)	G^- (noml/g)	G^+/G^-	DSE (noml/g)	Micro-shannon	Micro-simpson	Micro-evenness
2016	W	0.78	0.742	0.171	**$p < 0.05$**	0.756	0.257	0.477	0.774	0.329	0.657	0.329
	BW	0.211	0.249	**$p < 0.01$**	0.901	0.133	0.815	0.289	0.202	0.101	0.209	0.101
2017	W	0.349	0.324	**$p < 0.001$**	**$p < 0.01$**	0.146	0.34	0.384	0.172	**$p < 0.001$**	**$p < 0.01$**	**$p < 0.001$**
	BW	0.381	0.518	**$p < 0.001$**	**$p < 0.05$**	0.129	0.895	0.276	0.269	0.074	0.18	0.074

Figure S1. Effects of increased precipitation (W) and arbuscular mycorrhizal fungi suppression (BW) on relative abundance of microbial community components. CK: ambient precipitation treatment. G^{-}: Gram-negative bacteria; G^{+}: Gram-positive bacteria; Act: Actinobacteria; AMF: Arbuscular Mycorrhizal Fungi.

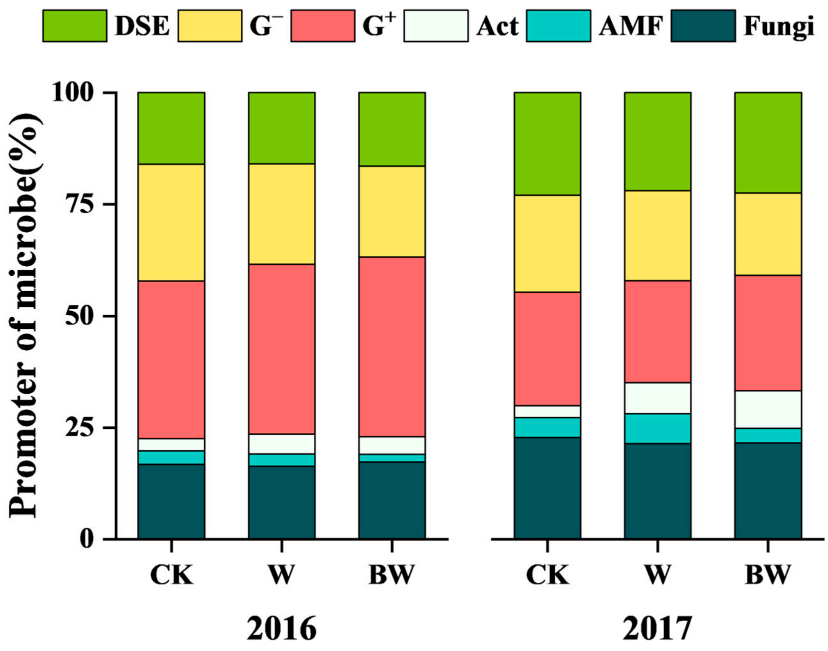

Figure S2. RDA Analysis of microbial communities on plant communities in 2016 (a) and 2017 (b). W: increased precipitation; BW: arbuscular mycorrhizal fungi suppression; CK: ambient precipitation treatment. G^-: Gram-negative bacteria; G^+: Gram-positive bacteria; Act: Actinobacteria; AMF: Arbuscular Mycorrhizal Fungi; G^+/G^-: The ratio of G^+ (PLFA) and G^- (PLFA); Total PLFA: Total PLFA content of microbial communities; DSE: Dark Septate Endophytes. *, $p < 0.05$; **, $p < 0.01$; ***, $p < 0.001$.

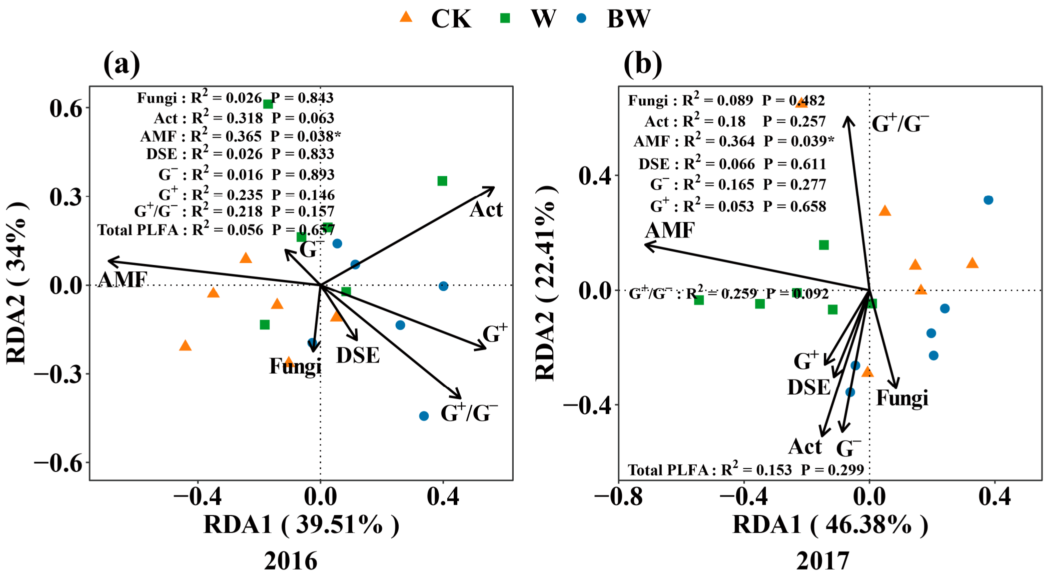

Figure S3. NMDS analysis of increased precipitation (W) and arbuscular mycorrhizal fungi suppression (BW) on microbial community structure in 2016 (a) and 2017 (b). CK: ambient precipitation treatment. *, p < 0.05; **, p < 0.01; ***, p < 0.001.

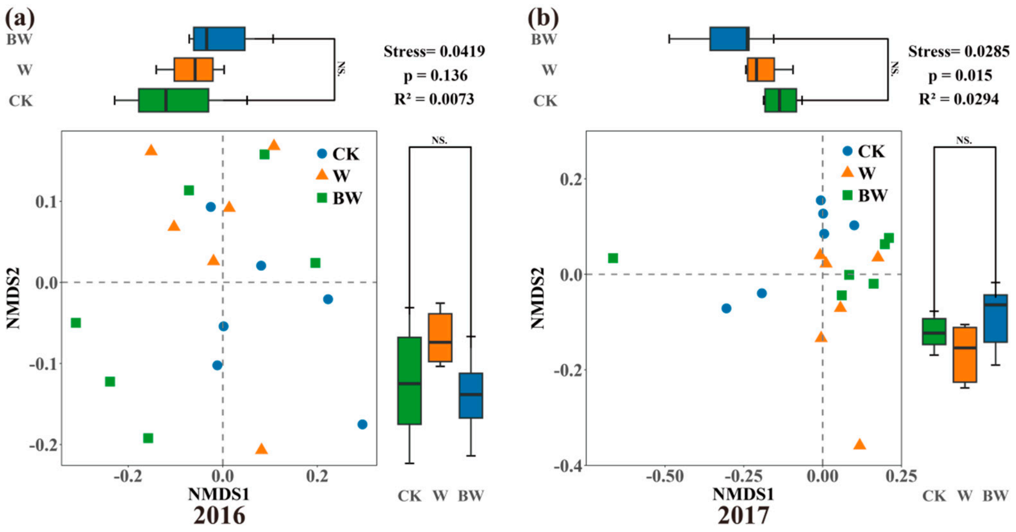

Figure S4. RDA Analysis of soil physicochemical properties on microbial communities in 2016 (a) and 2017 (b). W: increased precipitation; BW: arbuscular mycorrhizal fungi suppression; CK: ambient precipitation treatment. AP: Available Phosphorus; MBC: Microbial Biomass Carbon; MBN: Microbial Biomass Nitrogen; NH_4^+-N: Ammonium Nitrogen; NO_3^--N: Nitrate Nitrogen; SOC: Soil Organic Carbon. *, $p < 0.05$; **, $p < 0.01$; ***, $p < 0.001$.

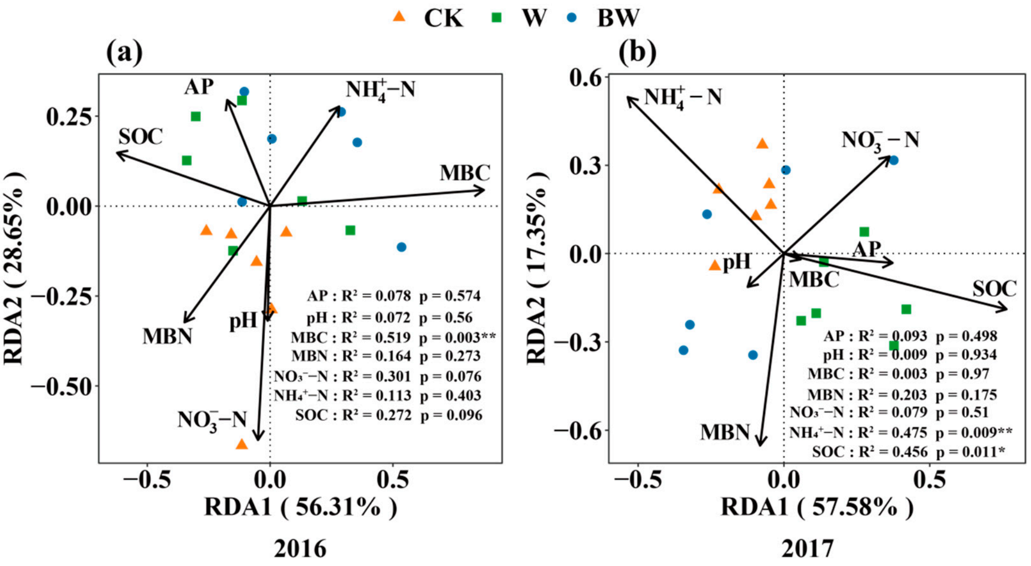

Figure S5. Relative importance of soil physicochemical properties for predicting the microbial community from random forest analysis. G^-: Gram-negative bacteria; G^+: Gram-positive bacteria; Act: Actinobacteria; AMF: Arbuscular Mycorrhizal Fungi; G^+/ G^-: The ratio of G^+ (PLFA) and G^-(PLFA); Total PLFA: Total PLFA content of microbial communities; DSE: Dark Septate Endophytes; AP: Available Phosphorus; MBC: Microbial Biomass Carbon; MBN: Microbial Biomass Nitrogen; NH_4^+-N: Ammonium Nitrogen; NO_3^--N: Nitrate Nitrogen; SOC: Soil Organic Carbon. *, $p < 0.05$; **, $p < 0.01$; ***, $p < 0.001$.

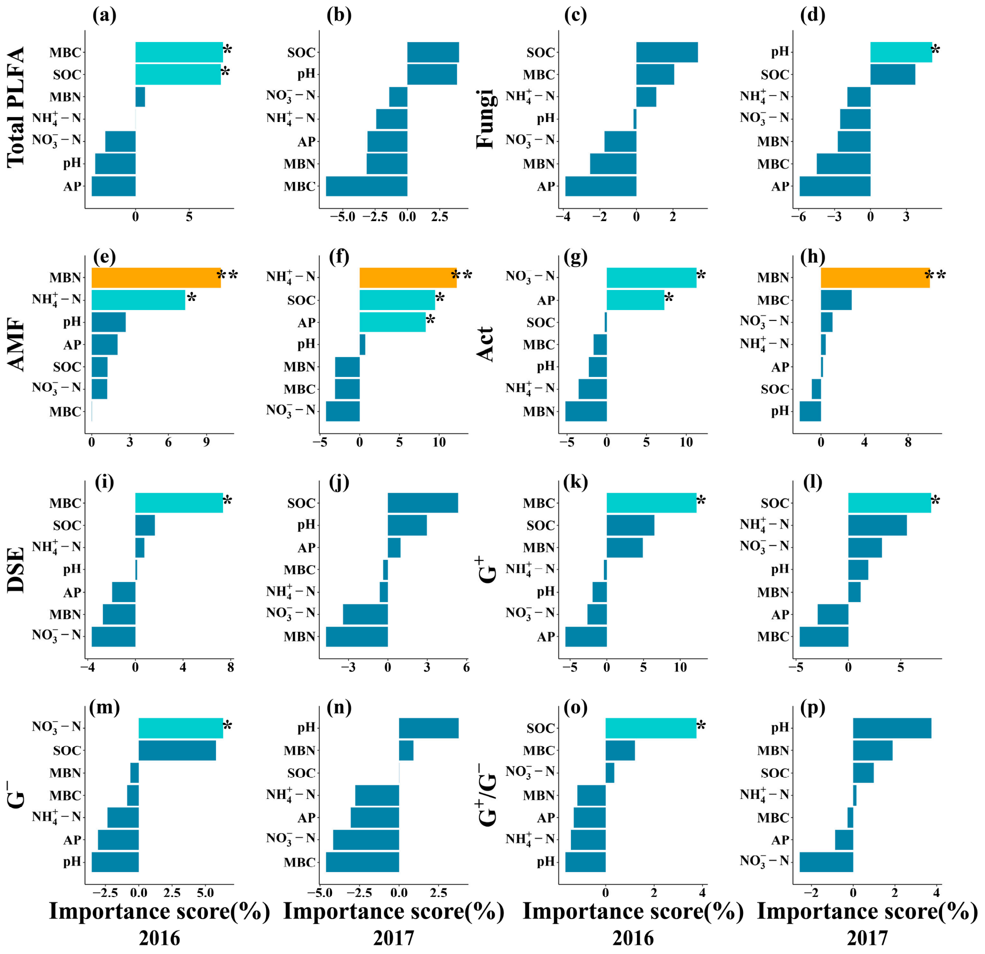

Supplement: Supplementary file 1 [file plants-14-03386-s001.zip › plants-3928830-supplementary.pdf]
